# Cone-like graphene nanostructures: electronic and optical properties

**DOI:** 10.1186/1556-276X-8-384

**Published:** 2013-09-12

**Authors:** Pablo Ulloa, Andrea Latgé, Luiz E Oliveira, Monica Pacheco

**Affiliations:** 1 Departamento de Física, Universidad Técnica Federico Santa María, Casilla 110-V, Valparaíso, Chile; 2 Instituto de Física, Universidade Federal Fluminense, Niterói, RJ 24210-340, Brazil; 3 Instituto de Física, Universidade Estadual de Campinas-UNICAMP, Campinas, SP 13083-859, Brazil

**Keywords:** Nanocones, Graphene, Optical absorption

## Abstract

A theoretical study of electronic and optical properties of graphene nanodisks and nanocones is presented within the framework of a tight-binding scheme. The electronic densities of states and absorption coefficients are calculated for such structures with different sizes and topologies. A discrete position approximation is used to describe the electronic states taking into account the effect of the overlap integral to first order. For small finite systems, both total and local densities of states depend sensitively on the number of atoms and characteristic geometry of the structures. Results for the local densities of charge reveal a finite charge distribution around some atoms at the apices and borders of the cone structures. For structures with more than 5,000 atoms, the contribution to the total density of states near the Fermi level essentially comes from states localized at the edges. For other energies, the average density of states exhibits similar features to the case of a graphene lattice. Results for the absorption spectra of nanocones show a peculiar dependence on the photon polarization in the infrared range for all investigated structures.

## Background

Since the first observation [1] of carbon nanocones (CNCs), large progress has been made on synthesis, characterization, and manipulation of CNCs and carbon nanodisks (CNDs) [2-6]. Differently from a planar graphene, the CNCs show a mixing of geometric, topological, and symmetry aspects that are exhibited in a non-homogeneous distribution of the electronic states through the structure. Particular effects of such feature are the charge accumulation at the cone apix and the selective polarized light absorption that may be used in technological applications.

There are different theoretical schemes to describe the electronic properties of cone-like structures. Models based on the Dirac equation [7,8] give a convenient insight of properties in the long wavelength limit. However, for finite-size graphenes, the longest stationary wavelength occurs in the border, and a correct description of the states near the Fermi level is given in terms of edge states [9,10]. The boundary conditions appearing when the nanosystems exhibit edges, such as the cases of nanoribbons, nanodisks, and nanorings, are quite well defined within a tight-binding formalism. Contrarily, in the continuum model, different approaches are followed to incorporate boundary conditions including the case of infinite mass [11] that have been critically examined and compared to tight-binding results. *Ab initio* models [12,13] are able to predict detailed features, but they are restricted to structures composed of a few hundred atoms due to their considerable computational costs. Calculations based on a single *π* orbital are able to describe the relevant electronic properties [14-16]. In that spirit, we calculate the electronic structure and optical spectra of CNDs and CNCs within a tight-binding approach. CNC-structured systems generated by pentagonal and heptagonal defects were previously studied using a Green function recursive method [14,17]. An interesting point to raise about the advantages of the tight-binding model is the fact that differently from the Dirac model, it is not essential to define two sublattices (A and B). For nanocones, this is a relevant point since for odd number of pentagons it is not possible to define the A/B sublattices.

**Figure 1:**
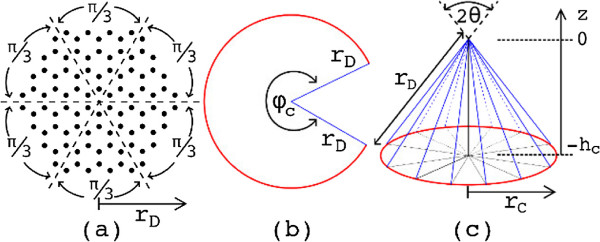
**Geometry elements.** (Color online) Pictorial view of **(a)** a carbon disk composed of six wedge sections of angle *π*/3, then **(b)** the removal of a $n_{w}=\{1,\ldots ,5\}$ wedge sections from the disk, and **(c)** by folding, it is constructed as a cone. Geometrical elements: generatrix *r*_*D*_, height *h*_*c*_, base radius *r*_*c*_, and apex opening angle 2*θ*, where sin*θ*=1−*n*_*w*_/6.

The total number *N*_*C*_ of carbon atoms in a cone structure may be estimated by dividing the cone surface area by half of the hexagonal cell’s surface,

(1) $$N_{C}=\;[\;4\pi /(3\sqrt{3})](1-n_{w}/6){\left(r_{D}/a_{\text{CC}}\right)}^{2}\;\;,$$

where the disclination number *n*_*w*_ corresponds to the integer number of *π*/3 wedge sections suppressed from the disk structure and *r*_*D*_ is the cone generatrix (see Figure 1). The nanocone disclination angle is given by *n*_*w*_*π*/3. For example, for *n*_*w*_=1 and *r*_*D*_=1 *μ*m, the CNC has ≈10^8^ atoms. By extracting an integer number *n*_*w*_ of *π*/3 sections from a carbon disk (cf. Figure 1), it is possible to construct up to five different closed cones. For *n*_*w*_=1, the cone angle is 2*θ*_1_=112.9°, corresponding to the flattest possible cone. In this case, *h*/*r*_*C*_=0.66 and *h*/*r*_*D*_=0.55.

In this work, finite-size systems (from 200 up to 5,000 atoms) are studied by performing direct diagonalizations of the stationary wave equation in the framework of a first-neighbor tight-binding approach. Each carbon atom has three nearest neighbors, except the border atoms for which dangling bonds are present. The overlap integral *s* is considered different from zero. As we will show later, this has important effects on the cone energy spectrum.

It is important to mention that relaxation mechanisms of the nanocone lattice are not explicitly included in the theoretical calculation. However, some stability criteria were adopted: (1) adjacent pentagonal defects are forbidden; (2) carbon atoms at the edges must have two next neighbors at least; (3) once the number of defects is chosen, the structures should exhibit the higher allowed symmetry (D6h group for the disk, D5 for the one-pentagon nanocone, and D2 for the nanocone with two pentagon defects). On the other hand, a statistical model to examine the feasibility and stability of nanocones has recently been reported [18]. Combined with classical molecular dynamics simulations and *ab initio* calculations, the results show that different nanocones can be obtained. An important result is that a small cone (consisting of only 70 atoms) is found to be quite stable at room temperature. One should remark that the nanosystems studied in the present work are composed with more than 5,000 atoms and an analysis based on *ab initio* methods of molecular dynamics should be prohibited.

Although some of the graphene electronic properties are present in the CNCs, deviations are always manifested as a consequence of the different atomic arrangements, the finite-size of the nanocones, and also the possible point symmetry of the distinct cones. In the absence of external fields, the calculated density of states (DOS) shows a peak at the Fermi energy, and the local density of states (LDOS) shows that electron states are localized at the cone base. On the other hand, the symmetries observed in the LDOS at different energies allow a systematic description of the electronic structure and selection rules of optical transitions driven by polarized radiation. Unlike the nanodisk, the presence of topological disorder in nanocones involves a deviation from the electrical neutrality at the apex and at the edges.

## Methods

In what follows, we present results for *n*_*w*_={0,1,2}, corresponding to CND and CNCs whose disclination angles are 60° and 120°. For those systems, the *sp*^2^ hybridization may be neglected. The electronic wave function may be written as

(2) $$|\Psi \rangle =\overset{N_{C}}{\underset{j=1}{\sum }}C_{j}|\pi_{j}\rangle \;\;,$$

where the |*π*_*j*_〉 denotes the atomic orbitals 2*p* at site ${\vec{R}}_{i}$. Note that the overlapping between neighboring orbitals prohibits the set |*π*_*j*_〉 to be an orthogonal basis. Only in the ideal case of zero overlap *s*=0, the coefficients $C_{j}^{0}$ in $|\Psi \rangle =\overset{N_{C}}{\underset{j=1}{\sum }}C_{j}^{0}|\pi_{j}^{0}\rangle$ might be considered equal to the discrete amplitude probability $\langle \pi_{j}^{0}|\Psi \rangle$ to find an electron at the *j*-th atom (described by the one electron state |*Ψ*〉). We use the *s*≠0 basis, |*π*_*j*_〉, to construct the eigenvalue equation and the $|\pi_{j}^{0}\rangle$ base to calculate the properties related to discrete positions. Of course, to relate both bases, it is required to know the $\langle \pi_{j}^{0}|\pi_{j}\rangle$ projection.

We define a *N*_*C*_×*N*_*C*_ matrix *Δ*^(1)^ relating the nearest neighboring atomic sites *i*,*j*,

(3) $$\Delta_{\text{ij}}^{\left(1\right)}=\left\{\begin{array}{cc} 1, & \;(i,j)\text{are n.n.}\\ 0, & \;\;\;\;\text{otherwise} \end{array},\;\right\}\;.$$

Similarly,

(4) $$\Delta_{\text{ij}}^{\left(0\right)}=\left\{\begin{array}{cc} 1, & \;\;\;\;(i=j)\\ 0, & \;\text{otherwise} \end{array},\;\right\}\;.$$

The *S* overlap matrix elements are then given by

(5) $$S_{\text{ij}}=\langle \pi_{i}|\pi_{j}\rangle =\Delta_{\text{ij}}^{\left(0\right)}+s\Delta_{\text{ij}}^{\left(1\right)}\;.$$

The hopping matrix elements of the tight-binding Hamiltonian $Ĥ={Ĥ}^{\left(\text{at}\right)}+\hat{V}$ are

(6) $$\langle \pi_{i}|\hat{V}|\pi_{j}\rangle =t\;\Delta_{\text{ij}}^{\left(1\right)}\;,$$

where *t* is the hopping energy parameter. Assuming the eigenvalue equation ${Ĥ}^{\left(\text{at}\right)}\;|\pi_{j}\rangle =\varepsilon_{2p}|\pi_{j}\rangle$, the atomic matrix elements are

(7) $$\langle \pi_{i}|{Ĥ}^{\left(\text{at}\right)}|\pi_{j}\rangle =\varepsilon_{2p}\left[\Delta_{\text{ij}}^{\left(0\right)}+s\Delta_{\text{ij}}^{\left(1\right)}\right]\;,$$

and

(8) $$\langle \pi_{i}|Ĥ|\Psi \rangle =\varepsilon \langle \pi_{i}|\Psi \rangle \;\;\;,\;\;1\leq i\leq N_{C}\;\;.$$

The resulting equation system may be written as a generalized eigenvalue problem $H\vec{C}=\mathrm{\varepsilon S}\vec{C}$, where the column vector $\vec{C}$ contains the coefficient *C*_*j*_,

(9) $$\left[\varepsilon_{2p}(\Delta^{\left(0\right)}+s\Delta^{\left(1\right)})+t\Delta^{\left(1\right)}\right]\vec{C}=\varepsilon \left[\Delta^{\left(0\right)}+s\Delta^{\left(1\right)}\right]\vec{C}.$$

The general solution may be expressed in terms of the auxiliary variables $\vec{C}(0)$ and *ε*(0), which satisfy

(10) $$t\Delta^{\left(1\right)}\vec{C}(0)=\varepsilon (0)\vec{C}(0)\;.$$

As $\vec{C}(0)$ also satisfies Equation (9), we obtain

(11) $$\varepsilon =\;[\;\varepsilon_{2p}+(1+s\varepsilon_{2p}/t)\varepsilon (0)]/[1+\mathrm{s\varepsilon}(0)/t]\;.$$

The orthogonality condition for the electronic states

(12) $$\langle \Psi^{k}|\Psi^{l}\rangle ={\vec{C}}^{\mathrm{k‡}}\;S\;{\vec{C}}^{l}=\delta_{k,l}\;$$

implies that

(13) $$\vec{C}=\frac{\vec{C}(0)}{\sqrt{{\vec{C}(0)}^{‡}\;S\;\vec{C}(0)}}\;.$$

For the calculation of the DOS, we use a Lorentzian distribution

(14) $$\text{DOS}(\varepsilon )=2\overset{N_{C}}{\underset{j=1}{\sum }}\delta (\varepsilon -\varepsilon^{j})\approx 2\overset{N_{C}}{\underset{j=1}{\sum }}\frac{\Gamma /\pi }{{\left(\varepsilon -\varepsilon^{j}\right)}^{2}+\Gamma^{2}}\;.$$

It is important to mention that, in *ab initio* calculations of carbon systems with edges, the atomic edges are passivated by hydrogen atoms. For graphene nanoribbons, the hydrogen passivation effects are better described when hybridized sigma-orbitals are considered [19]. However, for a single pi-orbital model, position-dependent hopping amplitude is usually adopted. In the case of armchair ribbons, a single correction at the carbon atoms layering at the dimmer positions of the edges sites is enough to obtain similar results to the density functional theory (DFT) calculations, while for zigzag nanoribbons, the agreement between electronic structures obtained from tight-binding models with no passivation and DFT models including H-passivation are remarkably good for energies next to the Fermi energy. Making a parallel to nanocone systems, we believe that passivation effects may be neglect in a first approximation and that the main characteristics of the electronic properties are preserved within this simple model.

The LDOS is calculated in terms of the discrete amplitude probability, $\langle \pi_{i}^{0}|\Psi^{j}\rangle$,

(15) $$\text{LDOS}({\vec{R}}_{i},\varepsilon )=2\overset{N_{C}}{\underset{j=1}{\sum }}|\langle \pi_{i}^{0}|\Psi^{j}\rangle {|}^{2}\;\delta (\varepsilon -\varepsilon^{j})\;\;,$$

where

(16) $$\langle \pi_{i}^{0}|\Psi^{j}\rangle =\left[\Delta^{\left(0\right)}+\frac{s}{2}\Delta^{\left(1\right)}\right]{\vec{C}}^{j}\;,$$

as it is shown in the subsection ‘Discrete position approach.’

The local electric charge (LEC) related to the *π* electrons is calculated by assuming that the other five electrons and the six protons of the carbon atom act as a net charge +*e*. Assuming zero temperature and the independent electron approximation, only the states 1≤*j*≤*n*_*F*_ will be occupied, where

(17) $$n_{F}=\left\{\begin{array}{ccc} N_{C}/2 & \;, & N_{C}\;\text{is even}\\ (N_{C}+1)/2 & \;, & N_{C}\;\text{is odd}\;. \end{array}\right)$$

Taking into account that the states below *n*_*F*_ contribute with −2*e* and the fact that the *n*_*F*_ state contribution depends on the parity of the number of atoms in the system, the LEC is written as

(18) $$\text{LEC}(R_{i})=e\left[1-2\overset{n_{F}}{\underset{j=1}{\sum }}|\langle \pi_{i}^{0}|\Psi^{j}\rangle {|}^{2}+\gamma |\langle \pi_{i}^{0}|\Psi^{n_{F}}\rangle {|}^{2}\right]$$

with *γ*=0 and 1, for *N*_*C*_ even and odd, respectively.

Optical absorption coefficients *α*_*ε*_(*ω*) are calculated by considering perpendicular ($\hat{\varepsilon_{⊥}}=\hat{\varepsilon_{x}},\hat{\varepsilon_{y}}$), and parallel ($\hat{\varepsilon_{∥}}=\hat{\varepsilon_{z}}$) polarizations, in relation to the cone axis,

(19) $$\alpha_{\hat{\varepsilon }}(\omega )\propto \frac{1}{\omega }\underset{i,j}{\sum }{\left|\langle \Psi^{i}|\;\hat{\varepsilon }\cdot \vec{p}\;|\Psi^{j}\rangle \right|}^{2}\delta \left[\varepsilon^{j}-\varepsilon^{i}-\hbar \omega \right]\;\;,$$

with *ε*^*i*,*j*^ corresponding to the energies of occupied and unoccupied states, respectively.

The oscillator strength may be written in terms of the spatial operators ($\hat{x}$, ŷ, and $\hat{z}$) [20], i.e.,

(20) $$\langle \Psi^{i}|{\hat{p}}_{z}|\Psi^{j}\rangle =\frac{m_{e}}{\mathrm{i\hbar}}(\varepsilon^{j}-\varepsilon^{i})\langle \Psi^{i}|\hat{z}|\Psi^{j}\rangle \;\;\;,$$

where $\langle \Psi^{i}|\hat{z}|\Psi^{j}\rangle$ is calculated to first order in s, using (30) of the subsection ‘Discrete position approach,’

(21) $$\langle \Psi^{i}|\hat{z}|\Psi^{j}\rangle ={\vec{C}}^{\mathrm{i‡}}\left[z+\frac{s}{2}\left(\Delta^{\left(1\right)}z+z\Delta^{\left(1\right)}\right)\right]{\vec{C}}^{j}\;.$$

### Discrete position approach

A discrete position scheme in terms of the $|\pi_{j}^{0}\rangle$ states was used to represent functions of the position given in terms of the atomic base, since they satisfy the same properties of the position states, i.e., orthogonality

(22) $$\langle \pi_{i}^{0}|\pi_{j}^{0}\rangle =\Delta_{\text{ij}}^{\left(0\right)}\;,$$

and completeness

(23) $$\overset{N_{C}}{\underset{k=1}{\sum }}|\pi_{k}^{0}\rangle \;\langle \pi_{k}^{0}|=\hat{1}\;$$

in a *N*_*C*_-dimensional subspace. The identity operator may also be constructed using the *s*≠0 base as

(24) $$\hat{1}=\underset{k,l}{\sum }|\pi_{k}\rangle {\left(S^{-1}\right)}_{\text{kl}}\langle \pi_{l}|\;,$$

with the *S*^−1^≈*Δ*^(0)^−*sΔ*^(1)^+*O*(*s*^2^) matrix being different from the *N*_*C*_×*N*_*C*_ identity matrix *Δ*^(0)^.

We take |*π*^0^〉 as the discrete position state and assume that the matrix elements $f_{\text{ij}}^{R}$ of position-dependent functions $f(\vec{R})$ are known in the *s*=0 representation,

(25) $$f_{\text{ij}}^{R}=\langle \pi_{i}^{0}|\hat{f}|\pi_{j}^{0}\rangle =f({\vec{R}}_{j})\;\Delta_{\text{ij}}^{\left(0\right)}\;.$$

Differently from the *f*^*R*^ matrices, *f* matrices in the *s*≠0 representation

(26) $$f_{\text{ij}}=\langle \pi_{i}|\hat{f}|\pi_{j}\rangle \;$$

are not diagonal. However, by performing the similarity transformation

(27) $$\langle \pi_{i}|\hat{f}|\pi_{j}\rangle =\underset{k,l}{\sum }\langle \pi_{i}|\pi_{k}^{0}\rangle \;\langle \pi_{k}^{0}|\hat{f}|\pi_{l}^{0}\rangle \;\langle \pi_{l}^{0}|\pi_{j}\rangle \;,$$

we may obtain the unknown *f* matrix in terms of the known *f*^*R*^ matrix, provided the transformation rule between the *π*^0^ and *π* bases is known. By assuming $\langle \pi_{i}^{0}|\pi_{j}\rangle =\bar{\alpha }\Delta_{\text{ij}}^{\left(0\right)}+\bar{\beta }\Delta_{\text{ij}}^{\left(1\right)}$, the *s*≠0 representation may be found. The coefficients $\bar{\alpha }$ and $\bar{\beta }$ are obtained by using the identity (23) into Equation (5),

(28) $$\overset{N_{C}}{\underset{k=1}{\sum }}\langle \pi_{i}|\pi_{k}^{0}\rangle \;\langle \pi_{k}^{0}|\pi_{j}\rangle =\Delta_{\text{ij}}^{\left(0\right)}+s\Delta_{\text{ij}}^{\left(1\right)}+O(s^{2})\;,$$

and, to first order in *s*, ($\bar{\alpha }=1$ and $\bar{\beta }=s/2$) we have

(29) $$\langle \pi_{i}^{0}|\pi_{j}\rangle =\Delta_{\text{ij}}^{\left(0\right)}+(s/2)\;\Delta_{\text{ij}}^{\left(1\right)}\;.$$

By replacing (29) in (27), one obtains

(30) $$f_{\text{ij}}={\left[f^{\left(R\right)}+\frac{s}{2}\left(f^{\left(R\right)}\Delta^{\left(1\right)}+\Delta^{\left(1\right)}f^{\left(R\right)}\right)\right]}_{i,j}+O(s^{2})\;\;\;$$

as the matrix elements of a position-dependent function in the *π*-base.

## Results and discussion

### Electronic density of states

**Figure 2:**
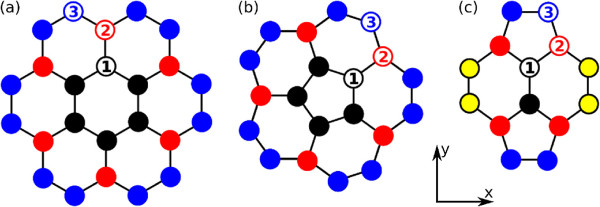
**Some relevant atomic sites.** Pictorial view of **(a)** a nanodisk center, **(b)** a one-pentagon nanocone apex, and **(c)** a two-pentagon nanocone apex. Atoms with different colors/numbers indicate different point symmetries for each system.

In what follows, we present numerical results for systems composed of up to 5,000 atoms. In the limit case of *N*_*C*_→*∞*, the energy spectrum is in the range from *ε*_min_=−3|*t*|/(1+3*s*) to *ε*_max_=+3|*t*|/(1−3*s*), the van Hove singularities occur at $\varepsilon_{\text{vH}}^{v}=-|t|/(1+s)$, $\varepsilon_{\text{vH}}^{c}=+|t|/(1-s)$, *t*=3 eV is the hopping integral and the Fermi energy is at *ε*_F_=0. A *Γ*=|*t*|/100 broadening and an overlap *s*=0.13 are assumed. In Figure 2, we show a pictorial view of the different studied systems in (a) a nanodisk center, (b) a one-pentagon nanocone apex, and (c) a two-pentagon nanocone apex. Atoms with different colors (numbers) indicate different point symmetries for each system.

**Figure 3:**
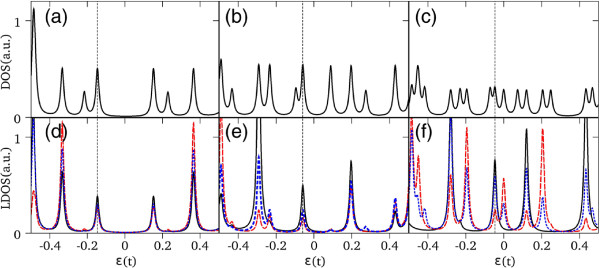
**Density of states for small systems.** (Color Online) DOS and LDOS for a *N*_*C *_= 258 nanodisk **(a,d)**, a *N*_*C *_= 245 one-pentagon nanocone **(b,e)**, and a *N*_*C *_= 246 two-pentagon nanocone **(c,f)**. LDOS curves for the different atoms shown in Figure 2, solid line (black atom 1), dashed line (red atom 2), and dotted line (blue atom 3). Vertical lines in each panel indicate the position of the Fermi energy.

Different plots in Figure 3 show the density of states averaged over the *N*_*C*_ atoms and the LDOS for a CND (Figure 3a,d), a single-pentagon CNC (Figure 3b,e), and for a two-pentagon CNC (Figure 3c,f), for *N*_*C*_=258,245, and 246, respectively. All results are shown in an energy range around *ε*_2p_=0.

As expected, for small finite systems, the DOS, LDOS, and the position of the Fermi energy depend on the number of atoms considered in the numerical calculation and on their characteristic geometries [21-23] and topology [24,25]. The experimental results by Ritter and Lyding [5] give actually a true conclusion about the influence of edge structure on the electronic structures of graphene quantum dots and nanoribbons. A remarkable difference between CND and CNCs structures is the existence of a finite DOS above the Fermi level for nanocones. This clear metallic character of the DOS for nanocones is more robust for the two-pentagon CNC [22,26]. This feature is a consequence of a symmetry break induced by the presence of topological defects in the CNC lattices, which generates new states above the Fermi energy not present in the CND structure. The contributions to the DOS coming from the apex atoms states are apparent in the LDOS of Figure 3e,f. Also notice that for the two-pentagon case, in which there is a large topological disorder, the LDOS spectra exhibit significant differences depending on the point symmetry of the considered atom (cf. Figure 2).

**Figure 4:**
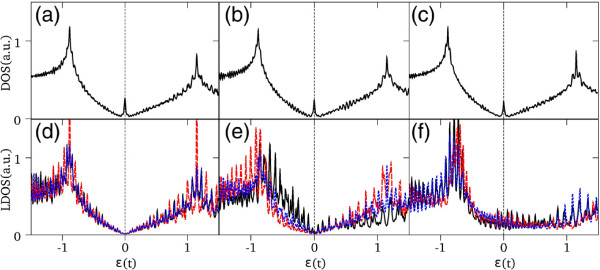
**Density of states for large systems.** (Color Online) DOS and LDOS for a *N*_*C *_= 5,016 nanodisk **(a,d)**, a *N*_*C *_= 5,005 one-pentagon nanocone **(b,e)**, and a *N*_*C *_= 5002 two-pentagon nanocone **(c,f)**. LDOS curves for the different atoms shown in Figure 2, solid line (black atom 1), dashed line (red atom 2), and dotted line (blue atom 3). Vertical lines in each panel indicate the position of the Fermi energy.

For increasing number of atoms, the total DOS for the different nanostructures is very similar to the corresponding DOS of a graphene layer, except for the edges states which show up as a peak at the Fermi energy, as shown in Figure 4a,b,c. It is interesting to note that the apex atomic states do not contribute to the total DOS near the Fermi energy but mainly near the graphene-like van Hove peaks. Notice that in the case of two-pentagon nanocones, the LDOS at the tip exhibits a robust metallic character.

**Figure 5:**
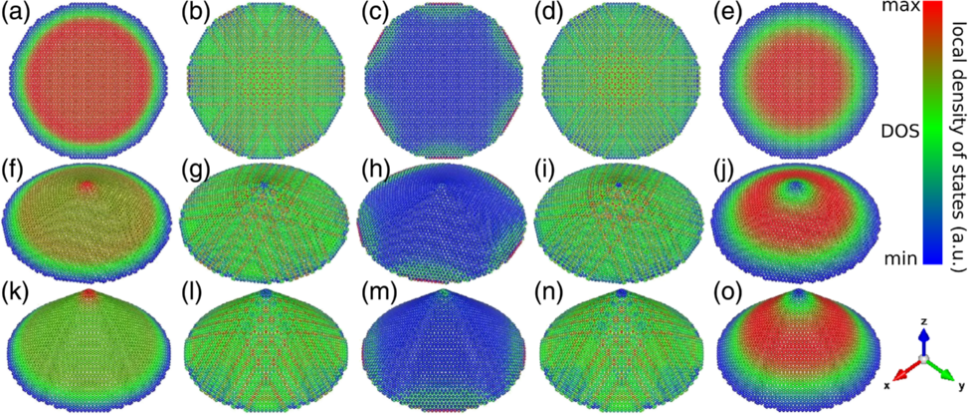
**Local density of states of the complete structures.** (Color Online) LDOS in arbitrary units for a 5,016-atom nanodisk **(a to e)**, a 5,005-atom nanocone with one pentagon at the apex **(f to j)**, and a 5,002-atom nanocone with two pentagons at apex **(k to o)**. The considered energies are (a,f,k) *ε*_min_, (b,g,l) $\varepsilon_{\text{vH}}^{v}$, (c,h,m) *ε*_F_, (d,i,n) $\varepsilon_{\text{vH}}^{c}$, and (e,j,o) *ε*_max_. The LDOS is measured with respect to the mean LDOS which is equal to the DOS at the considered energy.

To analyse the finite-size effects and the role played by the different symmetries of the cone-tip sites, we depict LDOS contour plots for the three studied structures by considering some characteristic energies: the minimum energy, the resonant peak below the Fermi energy, the Fermi energy, the resonant peak above the Fermi energy, and the maximum energy. Figure 5 illustrates the example of a CND with 5,016 atoms (top row), a single-pentagon CNC with 5,005 atoms (middle row), and a two-pentagon CNC with 5,002 atoms (bottom row). The electronic states corresponding to energies at the band extrema have the largest wavelength compared to the characteristic size of the system. In this way, the details of the lattice become less important and the states exhibit azimuthal symmetry. An interesting feature for the nanocones is that at these energies, the apex corresponds to a node for the maximum energy and an antinode for the minimum energy, respectively. On the other hand, the states at the Fermi energy are localized at the cone border, mainly at the zigzag edges as it is clearly shown in Figure 5c,h,m. For the states whose energy is near to the van Hove peaks, the LDOS reflects the symmetries of each system, i.e., for CND, the 2*π*/6-rotation symmetry and 12 specular planes (cf. Figure 5b,d), for a single-pentagon CNC, there is a 2*π*/5-rotation symmetry and five specular planes (cf. Figure 5g,i], and for a two-pentagon CNC, there is a *π*/2 rotation symmetry and two specular planes (cf. Figure 5l,i).

### Electric charge distribution

The electric charge per site, in terms of the fundamental charge *e*, was obtained using Equation (18). Results for the electric charge distribution for CNDs indicate that all the atomic sites preserve the charge neutrality, i.e., LEC = 0. For the CNCs, however, the atoms at the apex acquire negative charge and the atoms around the cone base exhibit positive charges at the zigzag edges. As *N*_*C*_ increases, the local electric charges at the apices, for the two studied CNC structures, tend to the asymptotic values shown in Table 1, which are in good agreement with the values reported by Green method calculations [14,17].

**Table 1:** **LEC (fundamental charge units) at some relevant atoms in the cone apices shown in Figure **2**b,c**

**Sites**	**1**	**2**	**3**	**Maximum**
One-pentagon	−0.071e	+0.014e	−0.059e	+0.042e
Two-pentagon	−0.055e	−0.067e	−0.066e	+0.076e

The maximum value occurs at the zigzag edge of each system.

**Figure 6:**
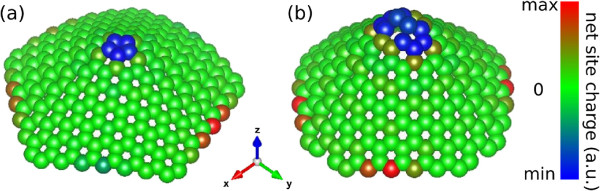
**Electric charge distribution in neutral CNCs.** (Color Online) For a single-pentagon cone with 245 atoms **(a)** and for two-pentagon cone with 246 atoms **(b)**. The values of electric charges for some sites are given in Table 1.

Figure 6 depicts the LEC for the two types of CNC structures, showing that the non-equilibrium of the charge distribution is restricted to the apex and edge regions: electric neutrality is found at all the other surface sites. The values found for the LEC at the apex regions are found to be independent of the size of the cones whereas this is not true for the edge states. When the number of atoms of the CNC structure is even, the edge-state LEC exhibits the same symmetry of the cone. For odd *N*_*C*_, the Fermi level is occupied by a single electron, and then, the LEC at the edge states reflects the breaking of symmetry.

### Absorption spectra

**Figure 7:**
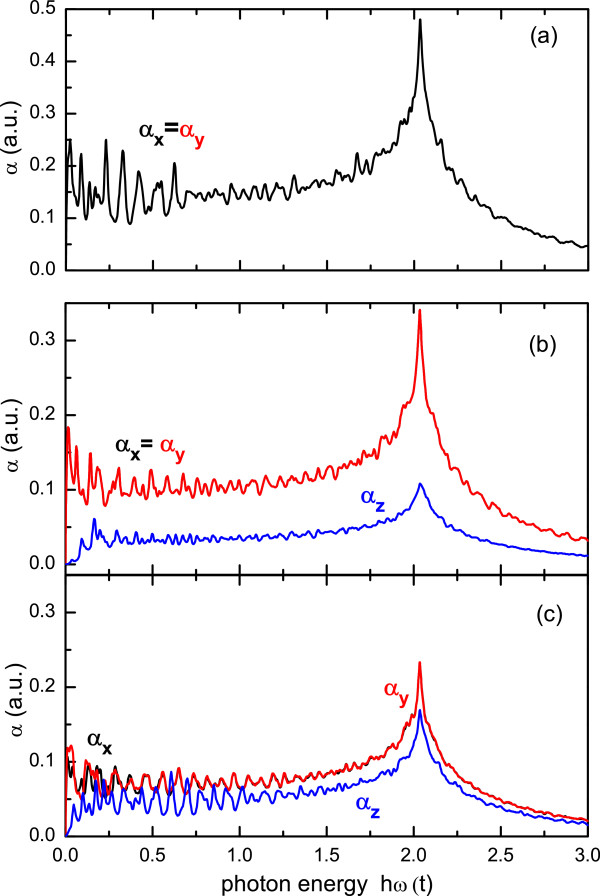
**Absorption spectrum for large systems.** (Color Online) Absorption coefficient for *x* (black curves), *y* (red curves), and *z* (blue lines) polarizations for **(a)** a nanodisk with 5,016 atoms, **(b)** a single-pentagon nanocone composed of 5,005 atoms, and **(c)** a two-pentagon nanocone with 5,002 atoms. The photon energies are given in units of ℏω/t.

We have also calculated the absorption coefficient for the CND and CNC structures, for different photon polarizations. Figure 7 shows the results for the absorption coefficients *α*_*x*_ and *α*_*y*_, for polarization perpendicular to the cone axis, and *α*_*z*_ for parallel polarization. Calculated results are shown for a nanodisk composed of 5,016 atoms, a single-pentagon nanocone with 5,005 atoms, and a two-pentagon nanocone with 5,002 atoms. For the case of large CNDs, the spectra present the general features observed for the absorption of a graphene monolayer. In the infrared region, the absorption coefficient of a graphene monolayer is expected to be strictly constant [27], whereas for higher energies the spectrum shows a strong interband absorption peak coming from transitions near the M point of the Brillouin zone of graphene [28]. The main difference for a finite CND is a departure from a completely frequency-independent behavior for low energies, where the absorption coefficient shows oscillations as a function of the photon energy instead of a constant value. This is a consequence of the border states that are manifested as a peak in the total DOS at the Fermi energy [24,29]. For CNCs, the general behavior is the same as for nanodisks, except for the dependence of the absorption on the photon polarization, in particular for low energies. Furthermore, the main absorption peaks for different polarizations occur when the photon energy is equal to the energy between the two DOS van Hove-like peaks (cf. Figure 4). Notice that the overlap integral *s*≠0 leads to an energy shift of the main resonant absorption peak given by *δ*≈2*s*^2^|*t*|/(1−*s*^2^)≈100 meV. This is a significant value for actual experimental measurements.

Concerning the different polarization directions, one should notice that, as occurs in *C*_6*v*_ symmetric systems, *α*_*z*_=0 and *α*_*x*_=*α*_*y*_ for the nanodisk. On the other hand, the absorption coefficients for the different cones studied (single and two pentagons) are finite for parallel polarization, and it depends on the structure details: as *α*_*z*_ increases for a two-pentagon CNC structure, *α*_*x*,*y*_ decreases. Due to the lack of *π*/2-rotation symmetry, one should expect, in principle, different results for x- and y-polarizations for any nanocone. However, such difference is observable just for the absorption coefficient of the two-pentagon CNC system, mainly in the range of low photon energies. The fact that *α*_*x*_=*α*_*y*_, for the case of one-pentagon CNC structure, may be explained using similar symmetry arguments applied to *C*_6*v*_ symmetry dots [24], extended to the *C*_5*v*_ symmetric cones. In the case of a two-pentagon CNC, the apex exhibits a *C*_2*v*_ symmetry, preventing the cone to be a *C*_4*v*_ symmetric system. As the apex plays a minor role, *α*_*x*_ and *α*_*y*_ will be slightly different. A large difference between the *α*_*z*_ and the *α*_*x*,*y*_ CNC absorption spectra occurs in the limit of low radiation energy. The *α*_*z*_ coefficient goes to zero as ℏω→0 whereas *α*_*x*,*y*_ shows oscillatory features. The behavior of the absorption for parallel polarization is due to the localization of the electronic states at the atomic sites around the cone border. As the spatial distribution of those states are restricted to a narrow extension along the *z* coordinate, the *z* degree of freedom is frozen for low excitation energies.

**Figure 8:**
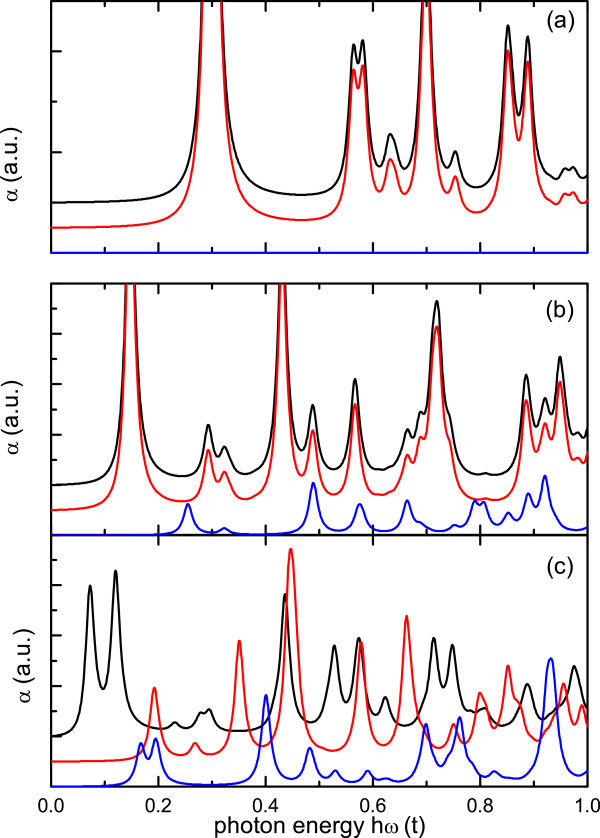
**Absorption spectrum for small systems.** (Color Online) Absorption coefficient for *x* (black curves), *y* (red curves), and *z* (blue lines) polarizations for **(a)** a nanodisk with 258 atoms, **(b)** a single-pentagon nanocone composed of 245 atoms, and **(c)** a two-pentagon nanocone with 246 atoms. The photon energies are given in units of ℏω/t. Curves in each panel are vertically shifted, for better visualization of different polarization results.

The dependence of the absorption spectra on the geometrical details of the different structures is more noticeable for finite-size nanostructures. This can be seen in Figure 8 which depicts the absorption coefficients for the CND composed of 258 atoms, the single-pentagon CNC with 245 atoms, and the two-pentagon CNC with 246 atoms. The degeneracy of the x- and y-polarization spectra is apparent for the smaller one-pentagon nanocone, as expected due to symmetry issues. On the other hand, the symmetry reduction for the two-pentagon structure leads to a rich absorption spectra, exhibiting peaks at different energies and with comparable weights for distinct polarizations. In that sense, absorption experiments may be an alternative route to distinguish between different nanocone geometries.

## Conclusions

Here, we have presented a theoretical study on the electronic properties of nanodisks and nanocones in the framework of a tight-binding approach. We have proposed a discrete position approximation to describe the electronic states which takes into account the effect of the overlap integral to first order. While the |*π*〉 base keeps the phenomenology of the overlap between neighboring atomic orbitals, the |*π*^0^〉 base allows the construction of diagonal matrices of position-dependent operators. A transformation rule was set up to take advantage of these two bases scenarios. Although the theoretical framework adopted does not explicitly include relaxation mechanisms, some stability criteria were adopted, and our analysis may be considered as a good first approximation to describe the main electronic structure and optical properties of such sizeable nanocones.

We have investigated the effects on the DOS and LDOS of the size and topology of CND and CNC structures. We have found that both total and local density of states sensitively depend on the number of atoms and characteristic geometry of the structures. One important aspect is the fact that cone and disk edges play a relevant role on the LDOS at the Fermi energy. For small finite systems, the presence of states localized in the cone apices determines the form of the DOS close to the Fermi energy. The observed features indicate that small nanocones could present good field-emission properties. This is corroborated by the calculation of the LEC that indicates the existence of finite charges at the apex region of the nanocones. For large systems, the contribution to the DOS near the Fermi level is mainly due to states localized in the edges of the structures whereas for other energies, the DOS exhibits similar features to the case of a graphene lattice.

The absorption coefficient for different CNC structures shows a peculiar dependence on the photon polarization in the infrared range for the investigated systems. The symmetry reduction of the two-pentagon nanocones causes the formation of very rich absorption spectra, with comparable weights for distinct polarizations. Although we have not found experimental data concerning to one-layer nanocones, we do believe that absorption measurements may be used as a natural route to distinguish between different nanocone geometries. The breaking of the degeneracy for different polarizations is found to be more pronounced for small nanocones. Absorption experiments may be used as natural measurements to distinguish between different nanocone geometries.

## Competing interests

The authors declare that they have no competing interests.

## Authors’ contributions

PU performed all the research and carried out the calculations. MP and AL supervised the work and drafted the manuscript. LEO revised the manuscript critically and provided theoretical guidance. All authors read and approved the final manuscript.
